# Process Kinetics of the Carbonation of Fly Ashes: A Research Study

**DOI:** 10.3390/ma14020253

**Published:** 2021-01-06

**Authors:** Marian Jacek Łączny, Sebastian Iwaszenko, Adam Smoliński

**Affiliations:** 1 Department of Solid Fuels Quality Assessment, Central Mining Institute, Plac Gwarków 1, 40-166 Katowice, Poland; jlaczny@gig.eu; 2 Department of Acoustics, Electronics and IT Solutions, Central Mining Institute, Plac Gwarków 1, 40-166 Katowice, Poland; 3 Central Mining Institute, Plac Gwarków 1, 40-166 Katowice, Poland; asmolinski@gig.eu

**Keywords:** fly ashes, carbonation, concrete admixtures, modeling, model parameter’s estimation

## Abstract

The aim of the paper is to present the results of research on the carbonation process kinetics of coal combustion ashes originating from fluidized bed boilers used in power plants. Based on the thermogravimetric analysis (TGA), the hypothesis that carbon dioxide is bounded by the mineral substances (calcium compounds) in the fly ashes was confirmed. Determining the kinetic parameters of the carbonation of fly ashes requires simultaneously taking into consideration the kinetics of the drying process of the sample. The drying process of the sample masks the effect of the reaction of CO_2_ with calcium compound. Unlike the ashes generated in pulverized fuel boilers, fly ashes contain irregular amorphic mineral components or poorly crystalized products of complete or partial dehydroxylation of claystone substance present in shale formations constituting the gangue as well as anhydrite (CaSO_4_), a desulfurization product. The content of free calcium oxide (CaO) in such ashes ranges from a few to several percent, which is a significant obstacle considering their use in cement and concrete production as type II admixtures understood to be inorganic grained materials of pozzolanic or latent hydraulic properties. The paper presents effective mechanisms which reduce the content of free CaO in ashes from Fluidized Bed Combustion (FBC) boilers to a level that allows their commercial utilization in the cement industry.

## 1. Introduction

The interest in circulating fluidized bed (CFB) boilers, including atmospheric circulating fluidized bed (ACFB) and pressurized circulating fluidized bed (PCFB) biolers, has been growing steadily due to their increasing combustion efficiency and considerably lower emissions of NO_x_ and SO_2_. In order to limit the emission of SO_2_, the admixture of limestone is required. Most commonly, two or three-fold surplus of CaCO_3_ in relation to the stoichiometric content of sulfur in coal is applied, enabling us to achieve over 90% reduction of SO_2_ emission [1,2]. At the same time, part of the CO_2_ generated in the combustion process can be utilized [3]. Thanks to the sufficient content of silica, alumina, calcium, and iron oxides, coal fly ash (CFA) is characterized by pozzolanic properties. Therefore, PFA has a wide range of applications in cement and concrete production [4,5]. In contrast, the CFA generated in pulverized fuel boilers is characterized by irregular amorphic mineral components or poorly crystalized products of complete or partial dehydroxylation of claystone substance present in shale formations constituting the gangue in addition to anhydrite (CaSO_4_), which is a desulfurization product. They also contain unreacted calcite (CaCO_3_), free CaO and Ca(OH)_2_—the product of its hydration as well as unreacted coal. While the management of CFA coming from pulverized fuel boilers does not pose a challenge, the utilization of CFA from fluidized bed boilers is problematic due to the high content of free CaO. The content of free calcium oxide in such CFAs ranges from a few to several percent, which results from the sorbent surplus. The application of the CFAs in cement and concrete production such as type II admixtures, understood as inorganic grained materials of pozzolanic or latent hydraulic properties, must comply with the European Standard EN 450-1:2005 [6] which specifies that they should not contain more than 2.5% of free CaO [6,7]. Moreover, calcium oxide is classified as a substance with irritating properties and has been labeled with GHS hazard statements H315 and H318 [8]. Waste is considered to be hazardous due to its irritating properties if the content of free CaO exceeds 1.0%. Waste from fluidized bed boilers which contains above 1.0 wt.% of free CaO may be classified as hazardous waste. It is also well known that the disposal of the waste at dumping facilities poses the risk of self-heating in the case of contact with water (a strong exothermic reaction) and, as a consequence, health and environmental hazards [9,10,11,12]. The content of CaO in FBC CFAs constitutes one of the most significant characteristics limiting their use as cement and concrete admixtures on a large scale. Relatively high content of free CaO as well as calcium sulfates (two components which have a considerable impact on the properties of cement mortar) coupled with the lack of sufficient amounts of silica, alumina, calcium, and iron oxides is the reason why they are not classified as ASTM admixtures of class F or C [13,14]. The occurrence of free CaO in in FBC coal fly ashes is connected with the mechanism of the calcination and desulfurization processes. It can also occur in the form of free grains or it may be built in the grain structure in connection with calcium sulfate [15]. There are two major procedures applied to remove CaO from the FBC coal fly ashes; one is the hydration reaction to calcium hydroxide [16], and the other is carbon dioxide sequestration including solid phases and aqueous solutions [17,18,19,20,21,22,23,24]. In recent years, there has been a growing interest in the application of limestone as filler [25]. Calcium carbonate is considered to be the key component influencing the processes of cement mortar binding. Treated as filler, calcite (CaCO_3_) (above 5%) performs the function of an active reagent. The mechanisms of the reactions are thoroughly discussed in previous researche [26,27,28]. Łączny suggested a new approach to the carbonation process [29]. In his study, the process of carbonation was used to achieve a product with a controlled content of free CaO. As a result, a three-component micro aggregate was obtained containing CaO_w_—CaCO_3_—CaSO_4_. The research confirmed that the aggregate may be used as an active component of cement mortars [30]. In addition, the study demonstrated a positive impact of carbonated FCB coal fly ashes on the strength of cement mortar. Łączny also demonstrated that the compressive strength of mortar made of FCB coal fly ashes is improved in comparison to non-carbonated ashes, which probably results from the positive impact of the carbonate on ettringite (Ca_6_Al_2_(SO_4_)_3_(OH)_12_·26H_2_O) transformation [31].

The aim of this paper is to present the results of research on the kinetics of carbonation process of coal combustion ashes from fluidized bed boilers used in power plants.

## 2. Materials and Methods

During the course of the study, the content of free calcium oxide in CFB coal fly ash was determined. The kinetics of the carbonation process occurring in the fluidized bed reactor was examined. The thermogravimetric analysis (TGA) was conducted by means of a TA Instruments differential scanning calorimeter (TA Instruments, New Castle, DE USA). Grain-size and mineralogical analyses of the waste were carried out using Malvern Morphologi G3S-ID analyzer. The determined sample volume was put into a ceramic container with a spatula and next placed in the chamber of the analyzer. The measurements were conducted under the following conditions: the speed of heating—5 °C/min, gas—60% CO_2_ in air, the temperature was kept constant for 15 min. The analyses were made at the temperatures of 40, 60, 80 and 100 °C.

Within the framework of the research, the chemical content of the CFA coming from fluidized bed boilers used in power plants was analyzed (see Table 1). In order to determine free CaO_w_, standard glycol method was applied. Next, grain-size analysis (granulometric analysis) of the waste samples was conducted using Malvern Morphologi G3S-ID analyzer with Raman chemical identification in order to examine the particle number, size and shape distributions. The dispersion parameters were as follows: dispersion pressure—0.8 bar, dispersion time—20.0 ms, setting time—60 s.

## 3. Results and Discussion

Table 2 shows the contents of free CaO_w_ in the analyzed waste samples. The content of free calcium oxide in CFA was determined as a function of time. On the day of the delivery of the analyzed waste, the content was 4.18% wt., whereas after 36 days of storage—0.5% wt. of CaO_w_ was missing, which led to the conclusion that the content of free calcium oxide changes during storage time, most probably as a consequence of the reaction of CaO with carbon dioxide present in the air.

The results of the grain-size analysis of the examined samples of CFA demonstrated that the content of coarse grains (100.0–1000.0 µm) in the sample was 0.011%, while the maximum grain diameter was 136.24 µm. The sample of CFA contained a greater amount of fine grains (1–10 µm)—88.4%, whereas the population of the finest grains (0.1–1 µm) was 5.38%. The content of 10.0–100.0 µm grains in the CFA samples was 6.18% (see Table 3).

### 3.1. Results of Thermogravimetric Analyses

**Figure 1:**
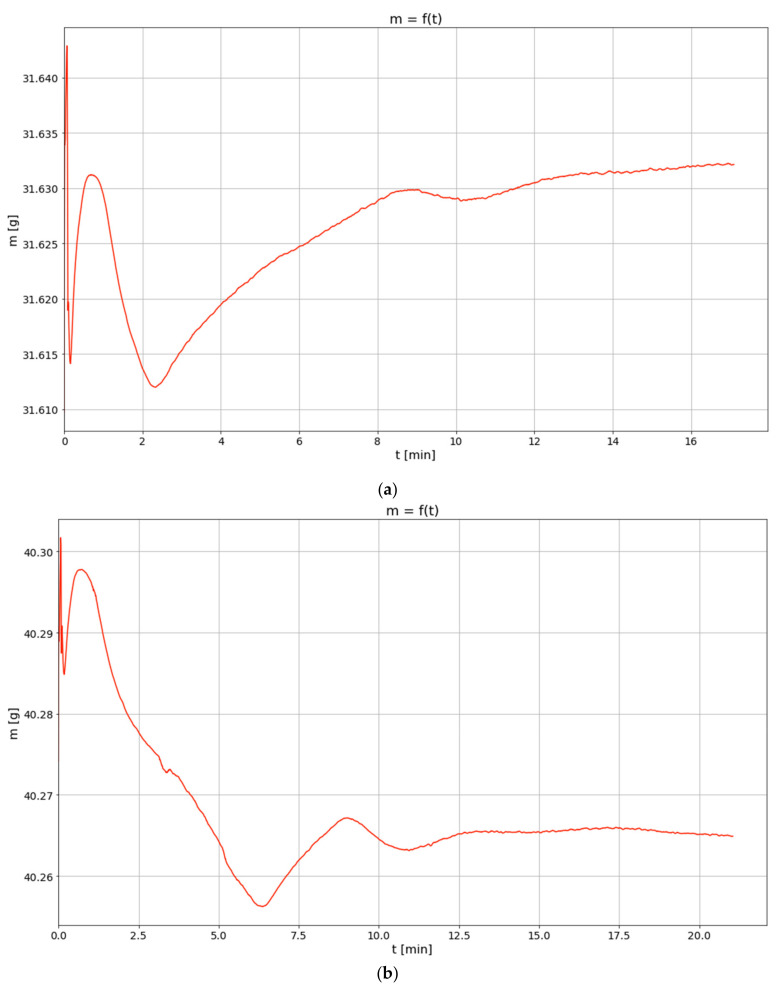

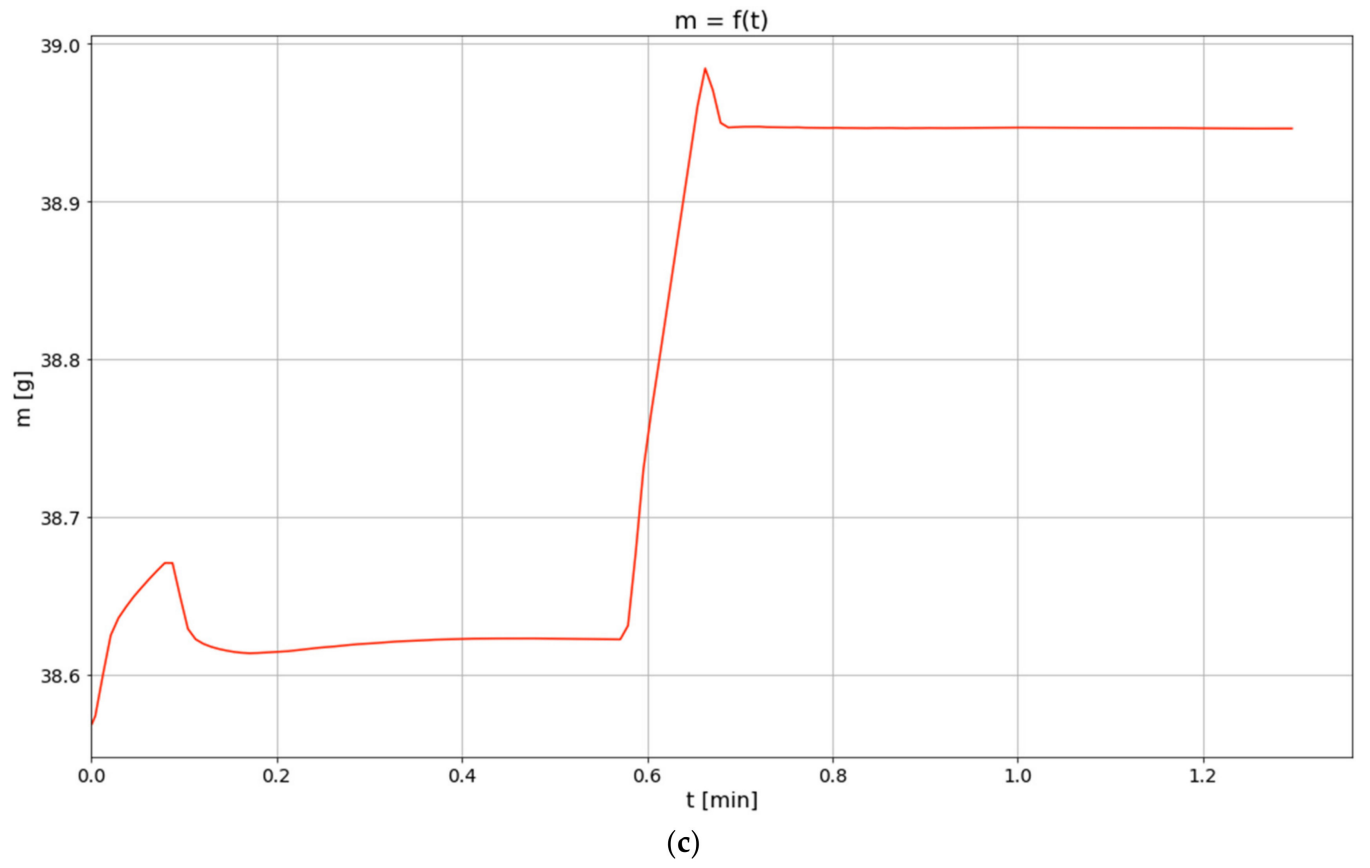
Thermogravimetric analysis of coal fly ash sample at the temperature of (**a**) 40 °C, (**b**) 60 °C and (**c**) 80 °C and in atmosphere of air + 60% CO_2_.

Figure 1 presents the results of thermogravimetric analyses. A few stages can be noticed in the thermograms; the first one lasts a few seconds and encompasses the initial phase of the research. It is connected with the streaming of the gas of a predetermined composition into the TGA chamber. This area of the thermogram does not include any data which would be essential for the analyses considering the discussed phenomena. The data gathered after the first maximum following the abrupt fluctuations of the sample mass should be taken into account. In the thermograms below, these values are assumed as reference values which serve to determine the sample mass loss during the second stage. Depending on a given sample, the loss ranged from about 0.06% (for the sample examined at the temperature of 40 °C) to 0.17% (for the sample examined at the temperature of 100 °C). This stage should be interpreted as the evaporation of surplus moisture from the sample. After this stage, for the samples examined in the lower range of temperatures (40, 60 and 80 °C, respectively), an increase in sample mass is observed. However, measurable values were recorded only for the first two cases. The increase may be regarded as the binding of CO_2_ by calcium compounds present in the structure of the analyzed material. In the case of the sample examined at the temperature of 100 °C, the mass increase is not observed, yet a significant decrease in the speed of the mass loss is noticeable. Such a course of the experiment suggests that, at lower temperatures, the rate of water evaporation is so low that it is possible to observe an increase in the sample mass resulting from the binding of CO_2_ in mineral structures. This interpretation is confirmed by the course of the process observed for the highest temperature. Because with the increase in temperature the binding of CO_2_ takes place with an increasingly higher rate, at a certain point this effect outweighs the mass loss resulting from the evaporation of water contained in the sample. In addition, poorly bound moisture is removed from the sample at the beginning of the process. When the process is progressing, the evaporation of another amount of water is connected with the necessity of spending additional energy to overcome the resistance of mass transport and the forces binding a water particle with mineral matter.

The conducted thermogravimetric analysis confirms the hypothesis that carbon dioxide is bound by the mineral substance (calcium compounds) contained in the analyzed waste samples. However, determining the kinetic parameters of the process based on TGA requires simultaneously taking into consideration the kinetics of the drying process of the sample because it may be masking the reaction of CO_2_ with calcium compounds.

### 3.2. The Use of a Dynamic Model Describing the Carbonation Process

The results shown in Figure 1 enables to present the carbonation process from a mathematical point of view. In each of the thermograms, the values of sample mass in the function of time can be observed. The charts represent a few consecutive phases, during which mass changes may be interpreted as the result of the physico-chemical transformations taking place in a given sample. Particular phases are especially noticeable for the temperatures of 40 and 60 °C, to a lesser extent for the temperature of 80 °C and only to a very little extent for 100 °C. The first phase of rapid mass changes taking place at the beginning of the experiment may be interpreted as an artefact indicating the occurrence of transient state which stabilizes after a short time. In the consecutive phase, a loss of mass occurs, which is explained by the evaporation of the sample moisture. The next phase, being a growth phase, is a manifestation of CO_2_ absorbance. This is the key phase for the discussed process and the research connected with the identification of model parameters has been limited to this very phase. Taking into account the obtained results, this phase is practically impossible to observe with regard to the data set for the temperature of 100 °C; therefore, this data set has been excluded from further considerations.

**Figure 2:**
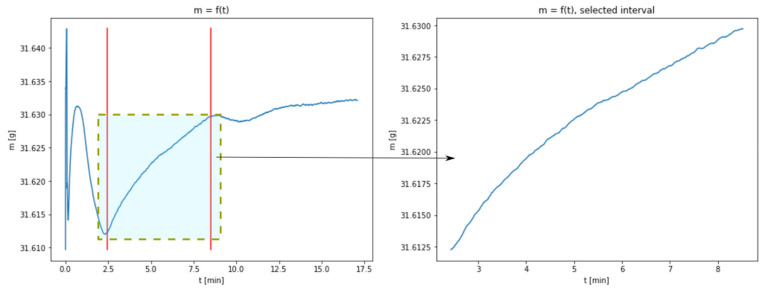
Data obtained from measurements at 40 °C with the isolated area used for model fitting (figure in the right presents the enlarged area).

**Figure 3:**
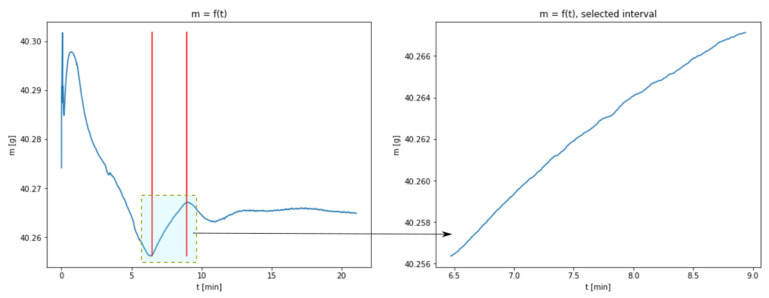
Data obtained from measurements at 60 °C with the isolated area used for model fitting (figure in the right presents the enlarged area).

**Figure 4:**
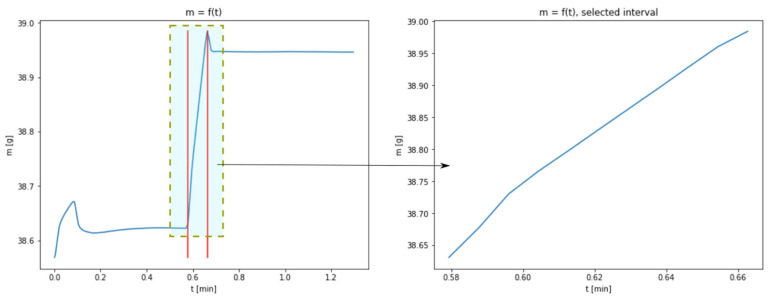
Data obtained from measurements at 80 °C with the isolated area used for model fitting (figure in the right presents the enlarged area).

The lack of a distinctive growth phase of the mass may be connected with a considerable acceleration of the carbonation process resulting from increased temperature. The complete transformation of CaO into CaCO_3_ takes place at the same time as the process of water evaporation; in the chart, a cumulative effect of both the phenomena is observed. A section related to the growth of sample mass was isolated from the charts representing temperatures of 40, 60 and 80 °C. The borders of the isolated areas were selected in such a way so as not to include the transient phases, i.e., the gradual flattening of the curve approaching the peak. Figure 2, Figure 3 and Figure 4 show the obtained charts.

The following carbonation equation was applied to formulate dynamic equations describing the loss of CaO at the time of the growth phase of sample mass resulting from the reaction with CO_2_:

(1) $$\mathrm{CaO}+{\mathrm{CO}}_{2}\to {\mathrm{CaCO}}_{3}$$

It was also assumed that the process takes place at the molecular level according to the accepted stoichiometry, and that there is a significant surplus of CO_2_ (its concentration does not change as a result of the reaction; it is a value constant in time). Based on this assumption, it is possible to formulate the following differential equation [32]:

(2) $$\frac{dN_{\mathrm{CaO}}}{dt}=-kN_{\mathrm{CaO}}$$

where *k* is the constant of reaction rate, t denotes time, whereas *N*_CaO_ is the number of CaO moles. This equation can be easily solved analytically to obtain the following relation:

(3) $$N_{\mathrm{CaO}}\left(t\right)=Ae^{-kt}$$

Constant *A* may be determined from the initial condition:

(4) $$N_{\mathrm{CaO}}\left(0\right)=N_{\mathrm{CaO}}^{0}=A$$

In the course of the carbonation reaction, CO_2_ molecule is bound to CaO. The resulting change of the sample mass may be described by means of the following reaction:

(5) $$\frac{d\Delta m\left(t\right)}{dt}=k\left(M_{{\mathrm{CaCO}}_{3}}-M_{\mathrm{CaO}}\right)N_{\mathrm{CaO}}\left(t\right)$$

$M_{{\mathrm{CaCO}}_{3}}$ denotes the molar mass of CaCO_3_, $M_{\mathrm{CaO}}$ stands for the molar mass of CaO, *d*m is the mass increase resulting from the pending carbonation process. Having applied Equation (3), we obtain:

(6) $$\frac{d\Delta m\left(t\right)}{dt}=N_{\mathrm{CaO}}^{0}\left(M_{{\mathrm{CaCO}}_{3}}-M_{\mathrm{CaO}}\right)\left(1-e^{-kt}\right)$$

While deriving the above equation, it was assumed that at the time t = 0 mass increase *d*m is zero. The number of CaO moles at the initial time can be associated with the initial CaO mass; thus, we obtain the final form of the equation:

(7) $$\frac{d\Delta m\left(t\right)}{dt}=m_{\mathrm{CaO}}^{0}\left(\frac{M_{{\mathrm{CaCO}}_{3}}}{M_{\mathrm{CaO}}}-1\right)\left(1-e^{-kt}\right)$$

The unknown quantities occurring in the equation, i.e., *k* and $m_{\mathrm{CaO}}^{0}$ may be determined using the experimental data. Because of *k* and $m_{\mathrm{CaO}}^{0}$, the following quality indicator was minimized:

(8) $$I=\underset{i\in I}{\sum }{\left[\Delta {\tilde{m}}_{i}-\Delta m\left(t_{i}\right)\right]}^{2}$$

**Figure 5:**
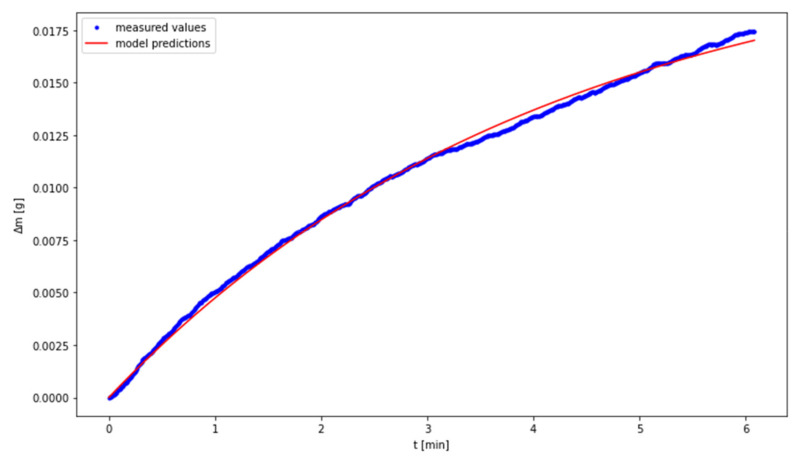
Fitting the model to experimental data for the temperature of 40 °C.

**Figure 6:**
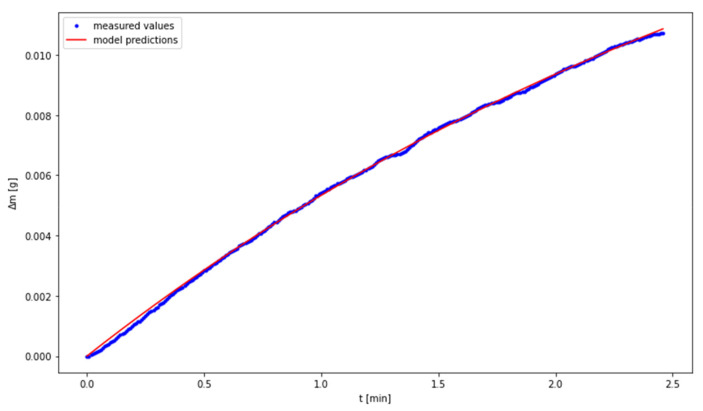
Fitting the model to experimental data for the temperature of 60 °C.

**Figure 7:**
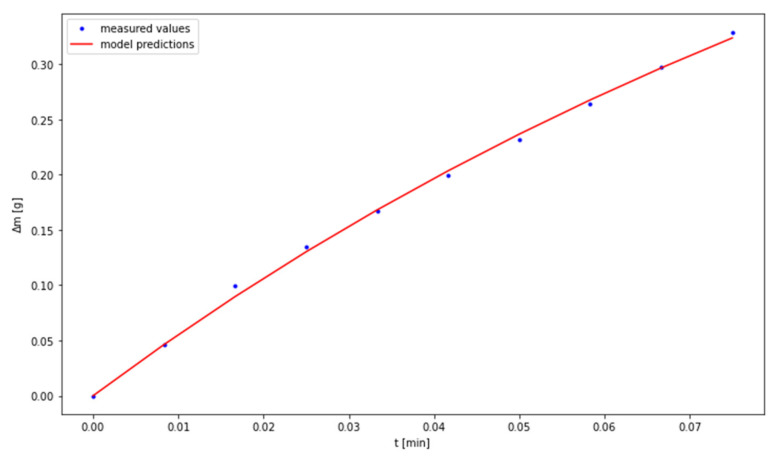
Fitting the model to experimental data for the temperature of 80 °C.

The fitting of the experimental data to the model was performed by means of Levenberg Marquardt algorithm. The results are presented in Figure 5, Figure 6 and Figure 7, while the values of the determined parameters are compiled in Table 4. With regard to the temperature of 80 °C, the outliers may be attributed to the small number of measurement points in the third phase of the discussed process.

The process of carbonation is a two-stage process which runs according to the following two exothermic reactions:

CaO + H_2_O = Ca(OH)_2_ (9)

Ca(OH)_2_ + CO_2_ = CaCO_3_ + H_2_O (10)

Adopting the assumption that the process takes place at the solid/gaseous phase contact seems to be justified based on the fact that the occurrence of free calcium oxide is associated with the mechanisms of the calcination and desulfurization; additionally, calcium oxide may assume the form of free grains or it may occur with calcium sulfate within the grain structure [33]. This is the reason why it is practically impossible to apply classic equations in order to describe the kinetics in a similar way to the approaches discussed in the field literature on the hydration and carbonation of pure sorbents [34,35,36,37]. The direct application of the approach according to which the process takes place in an aqueous environment is also impossible due to the prevalence of physical dissolution phenomena and the ionic reaction in liquid phase [38].

Bauer studied the carbonation process of lignite coal fly ashes under semi-dry conditions and at low temperatures and pressures [18]. His conclusion was that under such conditions, increasing the mixing intensity or the amount of CO_2_ also increases the carbonation rate. The reaction with CO_2_ depends on three factors, namely the dispersion of CO_2_ in the solid phase, the rate of Ca and Mg release from the mineral surface as well as the rate of the precipitation of carbonate solids; however, the reaction times in the semi-dry process route are considerably shorter. These observations confirm the results presented in this study. The presence of water in the sequence of follow-up reactions may be considered as one of the most significant factors influencing the rate of the process. In terms of practical applications as well as chemical engineering processes, it may facilitate the design of more efficient reactors in the future. A similar effect may be expected with reference to the impact of the bound CO_2_ on cement mortar hardening [39]. Our study confirmed that the application of a fluidized bed reactor filled with ceramic balls has a positive effect on the course of the carbonation process. As a result of the abrasion, the surface of the solid/gaseous phase contact enlarges additionally promoting the removal of products blocking the access to the reactants. The same effect was achieved in studies with the use of sonochemical treatment for the carbonation of circulating fluidized bed combustion (CFBC) coal fly ashes. Finally, CFA reactivity demonstrates strong dependence on temperature coupled with almost complete saturation of carbon dioxide at higher temperature ranges.

## 4. Conclusions

The conducted research confirmed the possibility of reducing the content of free calcium oxide in CFA from FBC boilers by treating them with carbon dioxide in the presence of water. In such a case, a reaction of carbon dioxide with free calcium oxide to calcium carbonate takes place and the progress of the reaction may be controlled so as to achieve a particular degree of calcium oxide conversion to calcium carbonate.

CFA reactivity demonstrates strong dependence on temperature coupled with almost complete saturation of carbon dioxide at higher temperature ranges. To determine the kinetic parameters of the carbonation of fly ashes, simultaneous analysis of the drying process of the ash sample must be analyzed. The drying process of the sample is masking the effect of the reaction of CO_2_ with calcium compound. Moreover, the most optimum conditions for the conversion of CFB waste to a product of controlled free CaO content were achieved using a fluidized bed reactor. With relatively short times of the reaction (30 min), a 2.7% wt. reduction of the calcium oxide content was obtained in comparison with the initial value.

## Figures and Tables

**Table 1:** Chemical composition of coal fly ash converted to oxides.

No	Ash Component	Content (wt.%)
1	SiO_2_	36.02
2	Al_2_O_3_	21.02
3	Fe_2_O_3_	6.41
4	CaO (including free CaO)	18.02 (4.18)
5	MgO	2.10
6	BaO	0.07
7	P_2_O_5_	0.33
8	Na_2_O	0.91
9	K_2_O	2.15
10	SO_3_	9.12
11	TiO_2_	0.63
12	LOI	2.55

**Table 2:** The content of free calcium oxide CaO_w_ in coal fly ash and in bottom ash.

Examined Samples	CaO_w_ (wt.%)
Fly ash—on the day of delivery	4.18
Fly ash after 18 days of storage	4.03
Fly ash after 36 days of storage	3.68
Bottom ash—on the day of delivery	2.76

**Table 3:** Systematic parameters of granulation.

Parameter	Unit	Value
Characteristic grain d	d^10^ * (µm)	1.23
	d^50^ * (µm)	2.86
	d^90^ * (µm)	7.60
Averaged diameter	(µm)	4.15
Standard deviation	(µm)	4.93

* d^10^, d^50^, d^90^—characteristic grain diameters, below which there occurs 10, 50, 90% of the analyzed material.

**Table 4:** The determined values of model parameters.

Constant	40 °C	60 °C	80 °C
m_0_ [g]	0.02815867	0.02773818	0.91305838
*k* [min^−1^]	0.24125613	0.28122913	8.02458336

## Data Availability

Data sharing not applicable.
